# Peruvian *chicha*: A Focus on the Microbial Populations of This Ancient Maize-Based Fermented Beverage

**DOI:** 10.3390/microorganisms8010093

**Published:** 2020-01-10

**Authors:** Daniela Bassi, Luigi Orrù, Jeison Cabanillas Vasquez, Pier Sandro Cocconcelli, Cecilia Fontana

**Affiliations:** 1 DISTAS, Università Cattolica del Sacro Cuore, via Emilia Parmense 84, 29122 Piacenza, Italy. Biotechnology Research Centre (CRB), via Milano 24, 26100 Cremona, Italy; daniela.bassi@unicatt.it (D.B.); pier.cocconcelli@unicatt.it (P.S.C.); 2 Consiglio per la Ricerca e la Sperimentazione in Agricoltura e l’Analisi dell’Economia Agraria, Centro di Ricerca per la Genomica Vegetale (CREA-GPG), 29017 Fiorenzuola d’Arda, Italy; luigi.orru@crea.gov.it; 3 Universidad Católica Sedes Sapientia, Esquina Constelaciones y Sol de Oro S/N, Urbanización Sol de Oro, Cercado de Lima 15302, Peru; jeijo.92.8@gmail.com; 4 INTA EEA Famaillá, Tucumán 4172, Argentina

**Keywords:** chicha de jora, maize, fermented beverage, NGS, bacterial communities

## Abstract

Peruvian chicha de jora is one of the most ancient traditional beverages produced through maize fermentation, still popular to modern consumers, but less studied in terms of microbial compositions. In this work, the bacterial biodiversity of 27 chicha samples collected from 14 different “chicherias” in seven provinces of Peru was investigated by Next-Generation Sequencing (NGS). A large dissimilarity in chicha microbial composition was a direct consequence of ingredients, manufacturing processes and geographical influences. The core microbiome was represented by six main genera, belonging to Lactic Acid Bacteria (LAB) and Acetic Acid Bacteria (AAB). Lactobacillus prevailed (more than 50% of sequences belong to this genus) followed by *Weissella*, *Leuconostoc, Lactococcus* and *Streptococcus*. Acetobacter was the only AAB genus identified in chicha. The occurrence of sequences associated to spoiling and pathogenic bacteria, such as *Bacillus*, *Clostridium*, and *Enterobacteriaceae*, was observed only in a few samples, validating the safety of this beverage. Predictive functional annotation of metagenomic sequences revealed that carbohydrate and amino acid metabolisms and coenzyme transport are the main KEGG categories associated to chicha fermentation pathways. The old recipes and traditional processing of each chicherias helps maintain native microorganisms as a resource of biodiversity with potential technological and health-beneficial properties.

## 1. Introduction

Andean populations deify maize as a magical and religious ingredient, primary compound of many traditional foods [1,2,3,4]. In particular, indigenous civilizations of Peru developed a fermented maize-based drink known as “chicha de jora”. This is a clear, yellowish, effervescent drink, with a low alcoholic content (1–3%) similar to a beer, consumed since pre-Hispanic times using primarily local maize varieties. This beverage, which is nowadays widely drunk in Peru, is made using the traditional technology as a direct continuation of a pre-Hispanic tradition and not yet industrialised. Traditionally, the production of chicha in Peru is carried out in familiar artisanal “chicherías”, and was mainly consumed by the native populations during religious social events and agricultural festivities [4]. The process begins with the preparation of corn malt obtained from “maiz morocho”, for whose elaboration the maize is soaked in water for a period of 3–8 days. Then, the water is drained and grains stay one or two weeks on a bed of leaves of *Sambucus nigra* (Sauco), *Baccharis latifolia* (Chilca), or *Alnus glutinosa* (Aliso) at room temperature, thus allowing their germination. After this, the sprouted grains (jora) are sundried for 1–2 weeks and then traditionally grounded by using stones. The obtained flour is added with water and boiled for one to two hours continuously sieving (on straw basket). At this step, as desired, other ingredients such as other kind of maize, “chancaca” (sugar cane tablets), sugar, barley, cloves, cinnamon, quinoa, wheat flour, fava beans, fruits, and herbs can be used. The fermentation occurs in different containers depending on chicherías facilities and is often an uncontrolled process that can last from 24h up to 15 days. [5]. According to old practices, ceramic vessels called “tomin” are used to start the fermentation process. The specific porous material of these containers helps the adherence and colonization of microorganisms. During the fermentation, small maize debris deriving from maize grounding, are continuously drained, rubbed and crumbled to obtain the “borra” that can be used as inoculum of microorganisms for the next productions. In fact, the addition of “borra de chicha”, followed by a second fermentation step, allows higher alcoholic level of the beverage.

Physiological and biochemical studies reported Lactobacillus and Leuconostoc genus as the main actors of the fermentation process [6,7]. More recently, molecular studies by next-generation sequencing (NGS) techniques revealed that lactic acid bacteria (LAB) and yeasts are the dominant populations responsible of the organoleptic traits of this beverage [8,9,10,11]. These data enlarged knowledge about the microbiota composition of this fermented product during the different steps of the manufacturing process, enabling the detection of minoritary species or of difficult-to-cultivate bacteria. Although maize-based chicha is produced in the Northwestern Argentina [9,11], cassava chicha from Ecuador [10] and mais or rice-based Brazilian chicha [12,13] have been investigated, thus no data are available for Peruvian chicha, where this beverage is still very popular.

In this study, the bacterial diversity associated with Peruvian chicha de jora produced in 14 chicherias located in seven different Peruvian provinces have been investigated, for the first time, by means of molecular techniques and next generation sequencing (NGS).

## 2. Materials and Methods

### 2.1. Samples Collection and DNA Extraction

Artisanal chicha samples (27) were collected from 14 different chicherias in 7 provinces of Perù (Table 1). 

Based on information collected by every local chicherias, controlled fermentations were considered those performed applying a more “standardized” processing in terms of recipes, containers (glass, plastic or earthen containers) and fermentation times, as reported in Table 1. Uncontrolled fermentation was generally made in random conditions such as open or closed containers, variable fermentation times and different recipes, and checked by the producer on the basis of their final taste. Samples of chicha, collected at retail stage and after vigorous mixing aimed to homogenize the product, were spotted onto Whatman^®^ FTA cards (Whatman, GE Healthcare, Little Chalfont, UK) and allow them to dry completely. The DNA was extracted from of FTA according to the manufacturer’s protocol.

### 2.2. Illumina 16S rRNA NGS

A high-throughput sequencing (HTS) approach using the Illumina Miseq platform (Illumina, San Diego, CA, USA) was applied on the 27 chicha samples. The bacterial V3-V4 16S rRNA region was amplified with the primer pairs 343F (50-TACGGRAGGCAGCAG-30) and 802R (50-TACNVGGGTWTCTAATCC-30) using the Phusion Flash High-Fidelity Master Mix (Thermo Fisher Scientific, Inc. Waltham, MA, USA), 0.5 µM of each primer and 0.1 ng of template DNA (25 µL final volume). A second PCR reaction was performed on the amplified fragments using tagged 343F primers according to the conditions previously described by Fontana et al. [14]. The PCR products for all samples were multiplexed in a single pool in equimolar amounts on the basis of the QuBit (Invitrogen Life Technologies, Waltham, MA, USA) quantification data. The PCR products pool was then purified using the solid phase reverse immobilization (SPRI) method of the Agencourt^®^ AMPure^®^ XP kit (Beckman Coulter, Italy, Milano) and sequenced at Parco Tecnologico Padano (Lodi, Italy). The TruSeq™ rDNA sample preparation kit (Illumina Inc., San Diego, CA, USA) was applied for the amplicon library preparation, whereas libraries were paired-end sequenced on Illumina MiSeq machine with 600 cycles (300 cycles for each paired reads).

### 2.3. Bioinformatic Analysis

Raw reads were processed to remove low quality reads using Trimmomatic applying a sliding window of 50 bp with an average quality of Q35 and a minimum read length of 120 [15]. The 16S rRNA sequences were assembled and analyzed using the Mothur software package version 1.39.5 [16]. For taxonomy-based analyses, sequences were aligned to the Greengenes database [17] and denoised to remove sequencing error. Chimera were removed using the Uchime algorithm [18] implemented in Mothur. Sequences were clustered into OTUs at 98% sequence identity using the opticlust method [19]. The sequences were classified using the references Ribosomal Database Project database (RDP) provided in Mothur. Alpha diversity and compositional analysis on chicha samples were performed using Microbiome Analyst software (McGill University, Montreal, QC, Canada) (https://www.microbiomeanalyst.ca/).

## 3. Results and Discussion

### 3.1. Chicha Samples Description

Among the traditional Andean maize-based fermented foods, chicha is “the ancient beer” that is routinely produced and consumed by Peruvian populations [5]. Nowadays, chicha prepared in urban areas is enriched with additional ingredients, other than “maiz morocho”, to vary aromas and to increase sweetness and alcohol content. Chicha samples, analyzed in this study, were collected from “chicherias” (markets, restaurants or “kioscos”) located in seven provinces, six belonging to Lima region and one to Ancash region. These beverages were basically produced following individual “chicherias” recipes, where the addition of a varieties of additives and with different fermentation times, resulted in a final product with peculiar traits. Table 1 shows the geographical area of production (province, region) of each sample and the fermentation conditions. Since the chicha way of production is still an artisanal practice that depends on handed down familiar traditions, the majority of samples were fermented following an uncontrolled process, whereas some samples were produced according to a controlled fermentation by means of earthen pots, covered buckets, or simply by tasting. Moreover, confirming the nonstandardized nature of this production, the fermentation times of the samples ranged from a minimum of 1 to a maximum of 7 days. Direct molecular analysis was performed on 27 chicha samples at the consumption stage collected locally. Since sample were mostly collected in areas where laboratory facilities are absent, the problem to preserve DNA for later analysis from degradation [20] was solved using FTA^®^ cards. Thus, we were able to extract DNA from samples spotted on these cards and perform on it microbial communities culture independent studies, as already reported [21,22,23].

### 3.2. High-Throughput Sequencing (HTS) Analysis

#### 3.2.1. Abundance Profiling

**Figure 1:**
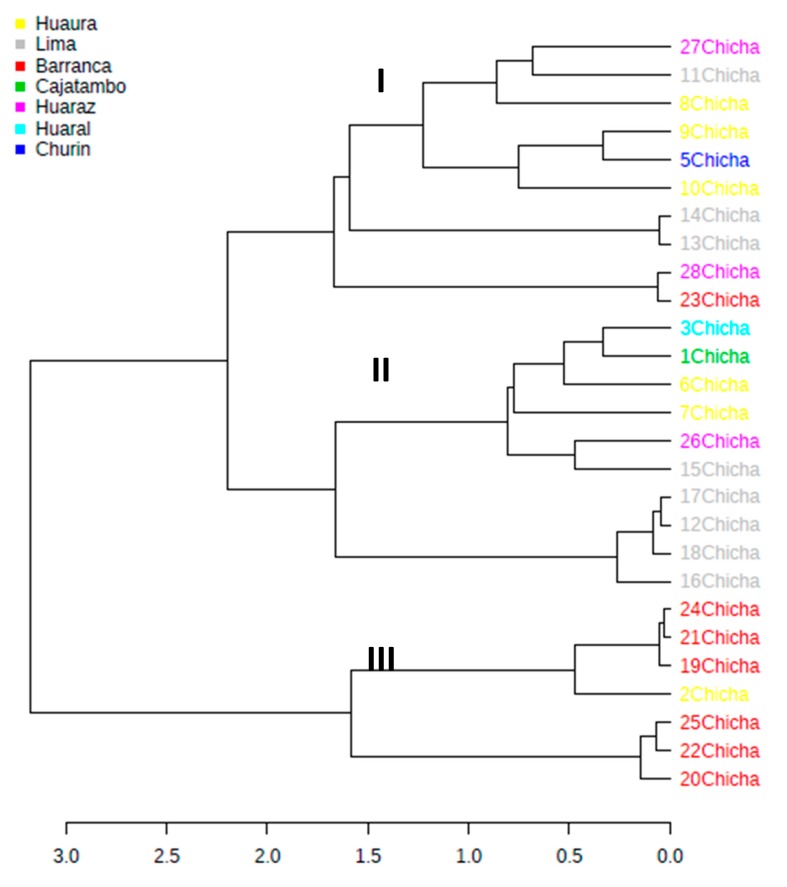
Cluster analysis of bacterial communities based on Bray–Curtis similarity. The analysis pattern includes all OTUs abundance data of the 27 chicha samples from the seven production provinces.

A deeper knowledge of the main autochthonous bacteria driving the fermentation of traditional chicha obtained without any standardized industrial process was assessed by a metabarcoding approach. In the current study, chicha samples produced in 27 different “chicherias” from seven Peruvian provinces (Table 1) were analyzed by sequencing the variable V3-V4 regions of the 16S rRNA gene. This work represents a comprehensive analysis of the bacterial populations of chicha to date, since data were generated from a larger collection of samples than has been studied heretofore. The analysis resulted in a total of 1,953,990 raw reads 300 bp in length, that passed quality filters and that were assigned to chicha microbiome samples. Rarefaction curves for each sample indicated that sequencing was deep enough to estimate the microbiome composition. A cluster analysis based on Bray–Curtis dissimilarity was used to compare the bacterial composition in chicha samples collected from the seven geographical areas. The dendogram obtained (Figure 1) shows that chicha samples were clustered in three main groups.

**Figure 2:**
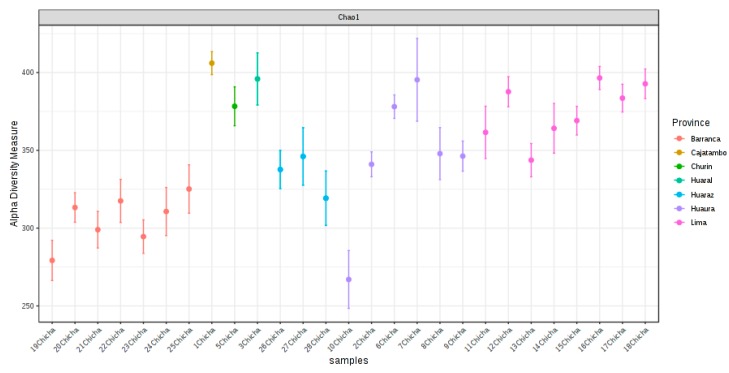
Plot of OTUs richness (Chao1) in all analyzed chicha samples. The Chao index estimate the total number of OTUs present in each chicha sample.

Cluster I was the most heterogeneous sector containing samples manufactured in Huaraz, Lima, Huaura, Barranca, and Churin; cluster II showed the presence of two subgroups, one associating only samples from Lima, whereas the other grouping chicha from different locations; cluster III contained almost all samples, except one, collected in Barranca; the presence of two distinct subgroups was also observed in this cluster. The large dissimilarity in microbial composition among samples described above was a direct consequence that chicha beverages were taken in different “chicherias” from the same city, indicating the importance of environmental influence and traditional recipes by individual producers on the chicha microbiota composition. Figure 2 shows the OTU richness (Chao index) calculated in individual samples, showing the heterogeneous profiles among samples collected in the same province.

**Figure 3:**
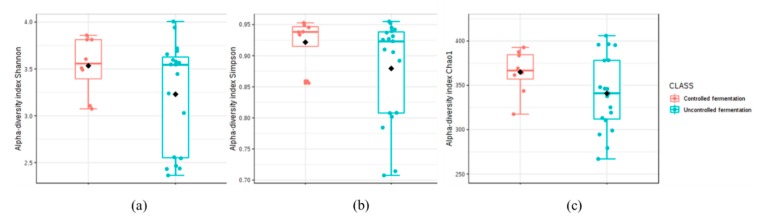
The Shannon (**a**), Simpson (**b**) and Chao1 (**c**) diversity indices of chicha communities regarding the controlled and uncontrolled fermentation experimental groups. The interquartile range is represented by the outer bounds of the boxes, the median is represented by the midline and the outliers are represented by the circles. The whiskers represent the minimum and maximum values.

The OTU richness showed to be on average values among samples from each province with the exception of Huaura that demonstrated the higher heterogeneity. Chao1 index, Shannon, and Simpson values used to determine the species diversity were also calculated taken into account the fermentation mode (Figure 3a–c).

These alpha-diversity numbers suggested a significantly higher diversity in bacterial populations of chicha samples with completely uncontrolled fermentation, than when the process was controlled for different parameters (Table 1). In this traditional beverage, the fermentation does not follow a standardized process but a spontaneous development of autochthonous microorganisms housing raw materials, handlings practices and environment [24] resulting in a completely randomized product both in terms of bacterial composition than of organoleptic properties.

#### 3.2.2. Bacterial Communities Profiling in Chicha Samples

**Figure 4:**
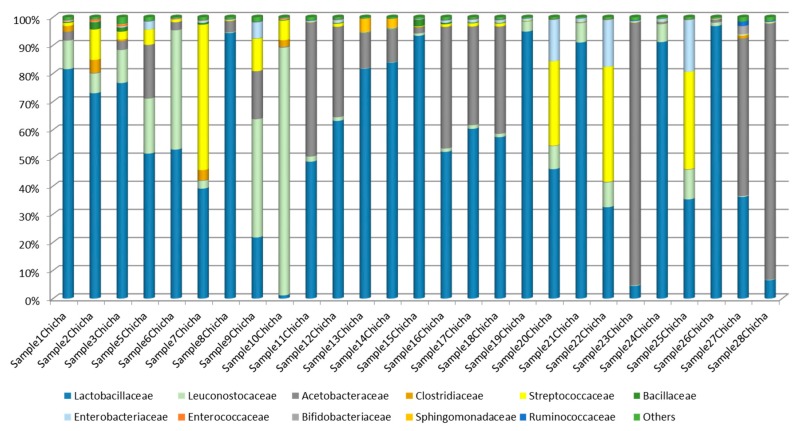
Bacterial community composition at family level of the 27 chicha samples.

Overall, taxonomical identification showed *Firmicutes* and *Proteobacteria* as the dominant phyla in the whole chicha samples; *Bacteroidetes* and *Actinobacteria* were also detected in some samples, although in small amount. Differences in bacterial taxonomic composition were more evident among samples when they were analyzed at family levels (Figure 4).

*Lactobacillaceae* and *Acetobateraceae* were the most representative families detected in the majority of analyzed chicha samples. In a total of 14 out of 27 samples more than 50% of the sequences were identified as *Lactobacillaceae*, whereas in samples 23Chicha, 27Chicha and 29Chicha *Acetobacteraceae* represented more than 50% of the sequences. *Leuconostocaceae* and *Streptococcaceae* were also detected in all samples, being dominating (more than 50%), respectively, in samples 10Chicha and 7Chicha. The other seven families of bacteria were found in small percentage in chicha samples: *Clostridiaceae, Bacillaceae, Enterobacteriaceae, Enterococcaceae, Bifidobacteriaceae, Sphingomonadaceae,* and *Ruminococcaceae*. In general, all the sample profiles at family level were almost different from one another.

**Figure 5:**
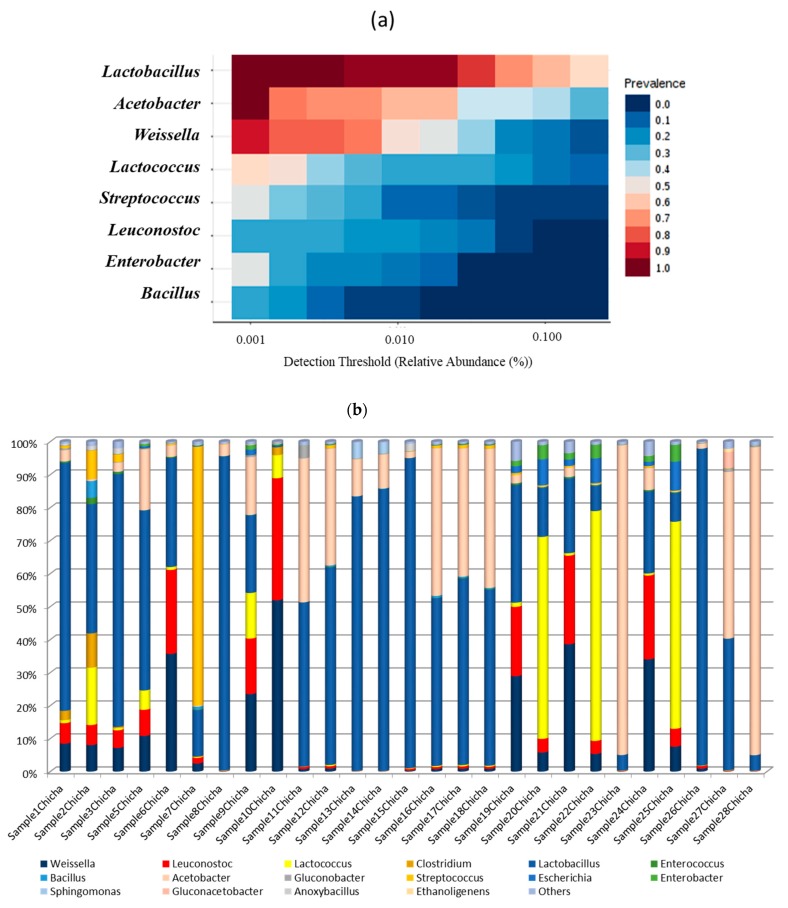
Core community (**a**) and distribution (**b**) at genus level of dominant bacteria colonizing *chicha* samples.

Despite the wide geographical area of sampling, the core community associated with chicha de jora produced in Peru, was represented by six main genera (Figure 5a) of which five belonged to the LAB group and only one to Acetic Acid Bacteria (AAB). The distribution and the relative abundance of the bacterial genera in each sample are illustrated in Figure 5b.

Among LAB genera, Lactobacillus prevailed (more than 50% of sequences belongs to this genus) followed by Weissella, Leuconostoc, Lactococcus, and Streptococcus. Acetobacter was the only AAB genus identified in chicha, being the most representative in three samples, confirming the family level distribution. A heterogeneous pattern of bacterial genera was observed in two chicha samples (9Chicha and 19Chicha), where the presence of a dominant microbial group wasn’t observed. The predominance of Lactobacillus in maize-based spontaneous fermentations has been largely described [9,25,26,27]. Particularly, Elizaquível et al. [9] investigating the main LAB genera present in chicha produced in the north of Argentina, reported that Enterococcus, Lactococcus, Streptococcus, Weissella, Leuconostoc, and Lactobacillus were the main genera identified by 16S metagenomics. Pyrosequencing of tagged 16S rRNA gene amplicons was also used to explore the bacterial microbiota in Colombian maize fermented dough “Masa Agria” describing a complex microbiota dominated by LAB and acetic bacteria [28]. Acetobacteraceae were found to be frequent bacterial colonizers in other maize-based fermented products such as the Mexican beverage Atole Agrio [29] and Mexican maize dough “Pozol” [1] and their presence in fermented vegetable food and beverages is widely reported [30].

The presence of Leuconostocaceae, already detected as secondary population in traditional vegetable fermented products [26,27,31], has been confirmed also in this study, where several chicha samples showed a high percentage of Leuconostoc and Weissella genus. Moreover, both genera were observed to be often present in equivalent proportions in the total microbial composition of each sample. The high content of carbohydrates in this maiz Morocho-based beverage represents an advantageous substrate for these LAB genera to produce non-digestible oligosaccharides and extracellular polysaccharides, mainly dextran, highly appreciated for their prebiotics properties [32].

**Figure 6:**
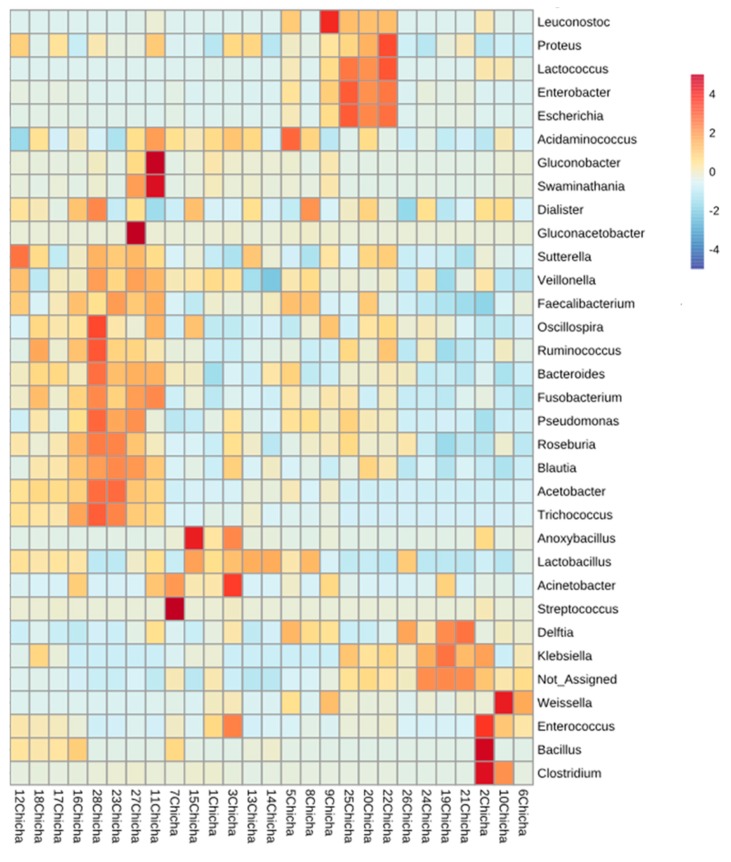
Heatmap showing the relative abundance of all OTUs of bacterial genus associated to chicha samples. Color keys represent square root of relative abundance (in number of sequences).

A more detailed picture of the most representative OTUs at genus level and their distribution in each chicha sample are represented in Figure 6.

In this heat map it is possible to observe how the OTUs identified reflect the fermentation trends as being different and sample specific. Beside the presence of the LAB core community, the presence of sequences associated to food-spoiling or pathogenic bacteria, such as Bacillus, Clostridium, and Enterobacteriaceae, was observed only in a few samples. LAB competitiveness in colonizing maize substrate and their ability to develop during the fermentation process could explain the low numbers of undesired bacterial development. When OTUs were investigated at species level (Table S1), 18 out of 26 were identified as originated from LAB species, being *Lactobacillus plantarum*, *Lactobacillus fermentum*, and *Weissella cibaria* the most largely found across the samples; sample *7Chicha*, the only one which showed the dominance of Streptococcus genus, contained OTUs belonging to *S. luteciae* and *S. alactolyticus*; among AAB, *Acetobacter okinawensis* was the prevalent species. Despite the natural and spontaneous fermentation processes to obtain Peruvian chicha, without standardized practices, LAB, largely recognized as beneficial and desired microorganisms, represent the main population in this Andean beverage.

The number of studies performed using Illumina technology on fermented foods has increased during the last decade, particularly those performed on milk and cheese derivatives and meat fermented products [33]. However, few studies using HTS technologies are used to investigate bacterial composition of traditionally produced vegetable-based foods in different Andean countries [9,28,29]. This is the first metagenomic analysis on several samples of indigenous Peruvian chicha from different areas and following ancient handed down recipes. Data obtained provide insightful information on the bacterial content of this typical Andean fermented beverage, revealing its diversity. The old recipes of each chicherias together with the traditional manufacturing techniques help to maintain native microorganisms as a resource of biodiversity.

### 3.3. Predictions of Metabolic Potentials

**Figure 7:**
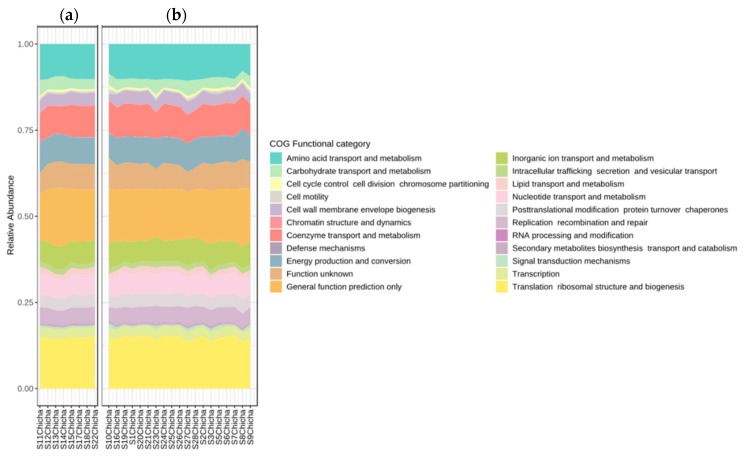
Functional categories defined by KEGG in chicha samples with (**a**) controlled fermentation and (**b**) uncontrolled fermentation.

Metagenomics of 16S and bioinformatics packages have become a powerful tool that allows both taxonomic and functional characterization of microbial communities. To determine the functional potential of the bacterial groups detected using 16S sequencing, we predicted the microbiome metagenome using PICRUST [34]. No significant differences in the functional categories defined by KEGG [35] were detected between the controlled and uncontrolled fermentation in all chicha samples (Figure 7).

A table containing relative KO abundance levels and KEGG functional categories is reported as Supplementary data (Tables S2 and S3). The predictive functional profiles of microbial communities revealed a relatively higher abundance of KEGG categories related to carbohydrate and amino acid metabolisms and coenzyme transport and metabolism, being these more abundant in samples containing high proportions of LAB.

## 4. Conclusions

In South America chicha de jora represents not only an ancestral tradition, but is nowadays a still widespread beverage around Andean countries because of its nutritional and healthy properties rather than just a tourist attraction. In this work, several chicha samples from different chicherias placed in Peru were investigated using HTS technologies helping us to understand that despite variations introduced by individual producers and recipes inherited through generations give rise to a product where fermentation is driven by a small number of genera dominated by LAB groups.

The data obtained in this study contribute to increase knowledge from a microbiological point of view of this kind of artisanal fermented product, where the presence of health promoting bacteria is important to determine its hygienic and beneficial health quality.

## Figures and Tables

**Table 1:** Geographical area of production and fermentation features of the 27 chicha samples.

Sample	Province	“Chicheria”	Region	Fermentation (days)	Additives	Note
1chicha	Cajatambo	Cajatambo	Lima	2	Yellow maize, sugar, “Chancaca”	Uncontrolled fermentation
2chicha	Huaura	“La Parada” Market	Lima	2	“Chancaca”, cinnamon (*Cinnamomum verum*), cloves (*Syzygium aromaticum*), barley (*Hordeum vulgare*), sugar	Uncontrolled fermentation
3chicha	Huaral	“La Parada” Market	Lima	3–4	Sugar, barley, wheat flour, quinoa (*Chenopodium quinoa*), fava beans, cinnamon, cloves	Uncontrolled fermentation
5chicha	Churin	Churin Central market	Lima	2	Barley, “Chancaca”	Uncontrolled fermentation
6chicha	Huaura	Huaura	Lima	1	Fava beans, barley, wheat flour, quinoa, orange banana, “Chancaca”	Uncontrolled fermentation
7chicha	Huaura	Huaura	Lima	3	Sugar, cinnamon, cloves	Uncontrolled fermentation
8chicha	Huaura	Huaura	Lima	4	Quinoa, barley, wheat, “Chancaca”	Uncontrolled fermentation
9chicha	Huaura	Huaura	Lima	2	Quinoa, roasted barley, wheat, “Chancaca”	Uncontrolled fermentation
10chicha	Huaura	Huaura	Lima	2	Sugar, roasted maize (“cancha serrana”) banana peel	Uncontrolled fermentation
11chicha	Lima	Restaurant “Caleta del Búho”	Lima	3–4	“Chancaca”	Controlled fermentation
12chicha	Lima	Lima	Lima	5	“Chancaca”, fava beans, quinoa, barley	Controlled fermentation
13chicha	Lima	Rimac	Lima	4	Sugar, barley, fava beans	Controlled fermentation
14chicha	Lima	Restaurant “Olla internacional”	Lima	7	Sugar	Controlled fermentation
15chicha	Lima	Restaurant “Costumbres Arequipeñas”	Lima	3	Chancaca, quinoa, fava beans	Controlled fermentation
16chicha	Lima	Restaurant “Tradiciones Arequipeñas”	Lima	3	Sugar, barley, fava beans, quinoa	Uncontrolled fermentation
17chicha	Lima	Restaurant “Semilla de Dioses”	Lima	4	Sugar, barley, fava beans, quinoa, *Chenopodium pallidicaule*	Controlled fermentation
18chicha	Lima	Restaurant “Sabor Andino”	Lima	3	Sugar, *Chenopodium pallidicaule*, fava beans, maca (*Lepidium meyenii*)	Controlled fermentation
19chicha	Barranca	Barranca	Lima	15	“Chancaca”, quinoa, maize amiláceo (*Zea mays* ssp *amiláceo*)	Uncontrolled fermentation
20chicha	Barranca	Barranca	Lima	3	Quinoa, cinnamon, cloves, “Chancaca”	Uncontrolled fermentation
21chicha	Barranca	Barranca	Lima	2	“Chancaca”, sugar, herbs	Uncontrolled fermentation
22chicha	Barranca	Barranca	Lima	4	White maize, quinoa, “Chancaca”, apple	Controlled fermentation
23chicha	Barranca	Barranca	Lima	3	Fava beans, quinoa, banana, cinnamon, “Chancaca”, cloves	Uncontrolled fermentation
24chicha	Barranca	Barranca	Lima	3	“Chancaca”, cinnamon, fava beans, cloves	Uncontrolled fermentation
25chicha	Barranca	Barranca	Lima	4	“Chancaca”	Uncontrolled fermentation
26chicha	Huaraz	Huaraz Market	Ancash	3	Fava beans, quinoa, “Chancaca”	Uncontrolled fermentation
27chicha	Huaraz	Huaraz Market	Ancash	2	Fava, white maize, “Chancaca”	Uncontrolled fermentation
28chicha	Huaraz	Huaraz Market	Ancash	2	“Chancaca”, quinoa	Uncontrolled fermentation

## Supplementary Materials

The following are available online at https://www.mdpi.com/2076-2607/8/1/93/s1, Table S1. Most abundant OTU at species level present in the 27 chicha samples. Table S2. Relative KEGG orthology groups (KO) and their abundance levels in chicha samples. Table S3. KEGG functional categories in chicha samples.

## References

[1] Wacher C., Canas A., Bä Rzana E., Lappe P., Ulloa M., Owens J.D. (2000). Microbiology of Indian and Mestizo pozol fermentations. Food Microbiol..

[2] Blandino A., Al-Aseeri M.E., Pandiella S.S., Cantero D., Webb C. (2003). Cereal-based fermented foods and beverages. Food Res. Int..

[3] Osorio-Cadavid E., Chaves-López C., Tofalo R., Paparella A., Suzzi G. (2008). Detection and identification of wild yeasts in Champús, a fermented Colombian maize beverage. Food Microbiol..

[4] Delibes R., Barragán A., Castillo Butters L.J., Bernier H., Lockard G., Rucabado Yong J. (2008). El Consumo Ritual de Chicha en San José de Moro. Arqueología Mochica: Nuevos Enfoques.

[5] Hayashida F.M. (2008). Ancient beer and modern brewers: Ethnoarchaeological observations of chicha production in two regions of the North Coast of Peru. J. Anthropol. Archaeol..

[6] Quillama E., Liendo N. (1995). Aislamiento e identificación de bacterias lácticas asociadas a Chicha de Jora. Bol. Lima.

[7] Steinkraus K.H. (2002). Fermentation in world food processing. Compr. Rev. Food Sci. Food Saf..

[8] Vallejo J.A., Miranda P., Flores-Félix J.D., Sánchez-Juanes F., Ageitos J.M., González-Buitrago J.M., Velázquez E., Villa T.G. (2013). Atypical yeasts identified as Saccharomyces cerevisiae by MALDI-TOF MS and gene sequencing are the main responsible of fermentation of chicha, a traditional beverage from Peru. Syst. Appl. Microbiol..

[9] Elizaquível P., Pérez-Cataluña A., Yépez A., Aristimuño C., Jiménez E., Cocconcelli P.S., Vignolo G., Aznar R. (2015). Pyrosequencing vs. culture-dependent approaches to analyze lactic acid bacteria associated to chicha, a traditional maize-based fermented beverage from Northwestern Argentina. Int. J. Food Microbiol..

[10] Freire A.L., Zapata S., Mosquera J., Mejia M.L., Trueba G. (2016). Bacteria associated with human saliva are major microbial components of Ecuadorian indigenous beers (*chicha*). PeerJ.

[11] Mendoza L.M., Neef A., Vignolo G., Belloch C. (2017). Yeast diversity during the fermentation of Andean chicha: A comparison of high-throughput sequencing and culture-dependent approaches. Food Microbiol..

[12] Puerari C., Magalhães-Guedes K.T., Schwan R.F. (2015). Physicochemical and microbiological characterization of chicha, a rice-based fermented beverage produced by Umutina Brazilian Amerindians. Food Microbiol..

[13] Resende L.V., Pinheiro L.K., Miguel M.G.d.C.P., Ramos C.L., Vilela D.M., Schwan R.F. (2018). Microbial community and physicochemical dynamics during the production of ‘Chicha’, a traditional beverage of Indigenous people of Brazil. World J. Microbiol. Biotechnol..

[14] Fontana C., Bassi D., López C., Pisacane V., Otero M.C., Puglisi E., Rebecchi A., Cocconcelli P.S., Vignolo G. (2016). Microbial ecology involved in the ripening of naturally fermented llama meat sausages. A focus on lactobacilli diversity. Int. J. Food Microbiol..

[15] Bolger A.M., Lohse M., Usadel B. (2014). Trimmomatic: A flexible trimmer for Illumina sequence data. Bioinformatics.

[16] Schloss P.D., Westcott S.L., Ryabin T., Hall J.R., Hartmann M., Hollister E.B., Lesniewski R.A., Oakley B.B., Parks D.H., Robinson C.J. (2009). Introducing mothur: Open-source, platform-independent, community-supported software for describing and comparing microbial communities. Appl. Environ. Microbiol..

[17] McDonald D., Price M.N., Goodrich J., Nawrocki E.P., Desantis T.Z., Probst A., Andersen G.L., Knight R., Hugenholtz P. (2012). An improved Greengenes taxonomy with explicit ranks for ecological and evolutionary analyses of bacteria and archaea. ISME J..

[18] Pérez-Losada M., Cabezas P., Castro-Nallar E., Crandall K.A. (2013). Pathogen typing in the genomics era: MLST and the future of molecular epidemiology. Infect. Genet. Evol..

[19] Westcott S.L., Schloss P.D. (2017). OptiClust, an Improved Method for Assigning Amplicon-Based Sequence Data to Operational Taxonomic Units. mSphere.

[20] Gray M.A., Pratte Z.A., Kellogg C.A. (2013). Comparison of DNA preservation methods for environmental bacterial community samples. FEMS Microbiol. Ecol..

[21] Kato H., Cáceres A.G., Mimori T., Ishimaru Y., Sayed A.S.M., Fujita M., Iwata H., Uezato H., Velez L.N., Gomez E.A.L. (2010). Use of FTA Cards for Direct Sampling of Patients’ Lesions in the Ecological Study of Cutaneous Leishmaniasis. J. Clin. Microbiol..

[22] Keeler S.P., Ferro P.J., Brown J.D., Fang X., El-Attrache J., Poulson R., Jackwood M.W., Stallknecht D.E. (2012). Use of FTA^®^ Sampling Cards for Molecular Detection of Avian Influenza Virus in Wild Birds. Avian Dis..

[23] Holt J.C., Purple K.E., Gerhold R. (2015). Use of FTA technology for detection of Trichomonas gallinae. Vet. Parasitol..

[24] Fábio Faria-Oliveira R.H.S.D., Fernanda Godoy-Santos F.B.P., Hygor Mezadri I.M.C., Brandão R.L. (2016). The Role of Yeast and Lactic Acid Bacteria in the Production of Fermented Beverages in South America. Food Production and Industry.

[25] Abriouel H., Ben Omar N., López R.L., Martínez-Cañamero M., Keleke S., Gálvez A. (2006). Culture-independent analysis of the microbial composition of the African traditional fermented foods poto poto and dégué by using three different DNA extraction methods. Int. J. Food Microbiol..

[26] De Vuyst L., Van Kerrebroeck S., Harth H., Huys G., Daniel H.M., Weckx S. (2014). Microbial ecology of sourdough fermentations: Diverse or uniform?. Food Microbiol..

[27] Schoustra S.E., Kasase C., Toarta C., Kassen R., Poulain A.J. (2013). Microbial Community Structure of Three Traditional Zambian Fermented Products: Mabisi, Chibwantu and Munkoyo. PLoS ONE.

[28] Chaves-Lopez C., Serio A., Delgado-Ospina J., Rossi C., Grande-Tovar C.D., Paparella A. (2016). Exploring the Bacterial Microbiota of Colombian Fermented Maize Dough “Masa Agria” (Maiz Añejo). Front. Microbiol..

[29] Pérez-Cataluña A., Elizaquível P., Carrasco P., Espinosa J., Reyes D., Wacher C., Aznar R. (2018). Diversity and dynamics of lactic acid bacteria in Atole agrio, a traditional maize-based fermented beverage from South-Eastern Mexico, analysed by high throughput sequencing and culturing. Antonie Leeuwenhoek.

[30] De Roos J., De Vuyst L. (2018). Acetic acid bacteria in fermented foods and beverages. Curr. Opin. Biotechnol..

[31] Jung J.Y., Lee S.H., Kim J.M., Park M.S., Bae J.W., Hahn Y., Madsen E.L., Jeon C.O. (2011). Metagenomic analysis of kimchi, a traditional Korean fermented food. Appl. Environ. Microbiol..

[32] Fusco V., Quero G.M., Cho G.-S., Kabisch J., Meske D., Neve H., Bockelmann W., Franz C.M.A.P. (2015). The genus Weissella: Taxonomy, ecology and biotechnological potential. Front. Microbiol..

[33] Cao Y., Fanning S., Proos S., Jordan K., Srikumar S. (2017). A Review on the Applications of Next Generation Sequencing Technologies as Applied to Food-Related Microbiome Studies. Front. Microbiol..

[34] Langille M.G.I., Zaneveld J., Caporaso J.G., McDonald D., Knights D., Reyes J.A., Clemente J.C., Burkepile D.E., Vega Thurber R.L., Knight R. (2013). Predictive functional profiling of microbial communities using 16S rRNA marker gene sequences. Nat. Biotechnol..

[35] Kanehisa M., Goto S., Sato Y., Furumichi M., Tanabe M. (2011). KEGG for integration and interpretation of large-scale molecular data sets. Nucleic Acids Res..

